# Luteolin alleviates depression‐like behavior by modulating glycerophospholipid metabolism in the hippocampus and prefrontal cortex of LOD rats

**DOI:** 10.1111/cns.14455

**Published:** 2023-09-16

**Authors:** Xiaofeng Wu, Hanfang Xu, Ningxi Zeng, Huizhen Li, Gaolei Yao, Kaige Liu, Can Yan, Lili Wu

**Affiliations:** ^1^ Integrative Medicine Research Center, School of Basic Medical Sciences, Guangzhou University of Chinese Medicine Guangzhou University of Chinese Medicine Guangzhou China; ^2^ Department of Rehabilitation Medicine, The People's Hospital of Longhua District Shenzhen China; ^3^ Key Laboratory of Depression Animal Model Based on TCM Syndrome, Key Laboratory of TCM for Prevention and Treatment of Brain Diseases with Cognitive Dysfunction Jiangxi University of Chinese Medicine Nanchang China

**Keywords:** autophagy disorder, glycerophospholipid metabolism, late‐onset depression, luteolin, metabolomics

## Abstract

**Background:**

Late‐onset depression (LOD) is defined as primary depression that first manifests after the age of 65. Luteolin (LUT) is a natural flavonoid that has shown promising antidepressant effects and improvement in neurological function in previous studies.

**Aims:**

In this study, we utilized UPLC–MS/MS non‐targeted metabolomics techniques, along with molecular docking technology and experimental validation, to explore the mechanism of LUT in treating LOD from a metabolomics perspective.

**Results:**

The behavioral results of our study demonstrate that LUT significantly ameliorated anxiety and depression‐like behaviors while enhancing cognitive function in LOD rats. Metabolomic analysis revealed that the effects of LUT on LOD rats were primarily mediated through the glycerophospholipid metabolic pathway in the hippocampus and prefrontal cortex. The levels of key lipid metabolites, phosphatidylserine (PS), phosphatidylcholine (PC), and phosphatidylethanolamine (PE), in the glycerophospholipid metabolic pathway were significantly altered by LUT treatment, with PC and PE showing significant correlations with behavioral indices. Molecular docking analysis indicated that LUT had strong binding activity with phosphatidylserine synthase 1 (PTDSS1), phosphatidylserine synthase 2 (PTDSS2), and phosphatidylserine decarboxylase (PISD), which are involved in the transformation and synthesis of PC, PE, and PS. Lastly, our study explored the reasons for the opposing trends of PC, PE, and PS in the hippocampus and prefrontal cortex from the perspective of autophagy, which may be attributable to the bidirectional regulation of autophagy in distinct brain regions.

**Conclusions:**

Our results revealed significant alterations in the glycerophospholipid metabolism pathways in both the hippocampus and prefrontal cortex of LOD rats. Moreover, LUT appears to regulate autophagy disorders by specifically modulating glycerophospholipid metabolism in different brain regions of LOD rats, consequently alleviating depression‐like behavior in these animals.

## Graphical abstract

In this study, we used UPLC–MS non‐targeted metabolomics techniques, combined with molecular docking technology and experimental verification, to explore the mechanism of LUT on LOD from a metabolomics perspective. These findings further improve the pathogenesis of LOD at the metabolomic level and provide new potential pharmacodynamic targets for LUT treatment of LOD.

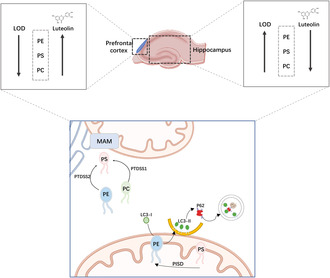

## 1 INTRODUCTION

Late‐onset depression (LOD) is a distinct subtype of geriatric depression, characterized by depressive disorders that first manifest at the age of 60 or later.^1^ It is one of the most prevalent mental health issues affecting the elderly population, with high incidence, mortality, and recurrence rates, coupled with low identification and treatment rates.^2^

The pathogenesis of LOD is multifactorial, with the hippocampus and prefrontal lobes playing important roles in associated neuropsychiatric disorders such as depression.^3^ Studies have demonstrated a reduction in hippocampal volume in LOD patients, with the extent of volume change correlating with the duration and severity of depression.^4^ Our previous research has also indicated a loss of mature neurons in the hippocampus with impaired neurogenesis in LOD rats.^5^ Additionally, the prefrontal lobe plays a significant modulatory role in negative emotions.^6^ As a key node of cortical and subcortical neural circuits, depressed patients exhibit a deficit in prefrontal cortical metabolism.^7^, ^8^, ^9^ Geriatric depression patients also display structural and functional loss in the prefrontal cortex compared to healthy older adults.^10^

Currently, the clinical pharmacotherapy for LOD often involves monotherapy or combination therapy. However, due to factors associated with aging, the drug's effectiveness decreases with age,^11^ and more than 50% of elderly patients do not respond to current antidepressants,^12^ making the drug treatment of LOD challenging. Luteolin (LUT) (3′,4′,5,7‐tetrahydroxyflavone) is a flavonoid commonly found in plant‐based foods and medicinal plants used in traditional medicine, with a variety of biological and pharmacological activities. Studies have shown that LUT has cognitive effects in animal models of cognitive deficits induced by high‐fat diets or aging, mainly related to its pharmacological anti‐inflammatory properties.^13^, ^14^ LUT also improves a variety of depression‐like behaviors through different pathways.^15^, ^16^, ^17^ Our previous studies have suggested that LUT can enhance the brain's transport of folic acid to promote hippocampal neurogenesis and alter the axonal guidance pathway in cerebrospinal fluid to exert its therapeutic effect on LOD.^5^, ^18^

Metabolomics technology is considered a valuable tool in the study of central nervous system diseases. Previous metabolomic studies on depression using plasma,^17^ brain tissue,^19^ and urine^20^ have been reported, revealing various metabolic pathways, such as amino acids,^21^ lipids,^22^ and energy metabolism^23^ that are associated with the onset of depression. Research indicates that Chinese herbs and natural products can regulate lipid metabolism in various disorders, including depression. Traditional herbal extracts, such as Pueraria Mirifica,^24^ Icariin,^25^ and Yanhuisuo alkaloids^26^ have been found to reverse depressive‐like behaviors by modulating lipid metabolism disorders, leading to a tendency of differential metabolite levels to return to normal, similar to the effects of positive drugs. Additionally, rhubarb has shown significant improvement in chronic kidney disease in rats, mitigating kidney function loss, and abnormal PS metabolism.^27^

Furthermore, aging of the body is inevitably accompanied by systemic metabolic changes, and the disorder of lipid metabolism is a characteristic of aging.^28^ Therefore, analyzing endogenous small molecule metabolites and the metabolic pathways involved has significant guiding significance in exploring the relationship between metabolites and physiological and pathological changes in LOD rats, as well as the drug treatment mechanism of LUT. In view of this, we conducted a metabolomics study of LUT on LOD to characterize global metabolic profile alterations in the brain and peripheral serum of rats during antidepressant drug studies.

In this study, we established a 6‐week chronic unpredictable mild stress (CUMS) model in naturally aged rats aged 20–21 months. We focused on the hippocampal and prefrontal cortex regions, as well as peripheral serum, to explore whether the antidepressant and anti‐aging effects of luteolin were localized in specific regions.

By comparing the levels of metabolites in the hippocampus, prefrontal cortex, and serum of rats in the LOD, CON, and LUT groups, we identified the pathway of action of LUT based on centrally and peripherally enriched metabolic pathways, revealing the metabolomic changes associated with stress and aging in LOD rats. Based on the results of metabolomics analysis, we conducted molecular docking of key metabolite synthases and LUT to explore the mechanism of inconsistent metabolite change trends in different brain regions of LUT from the perspective of the autophagy pathway.

## 2 MATERIALS AND METHODS

### 2.1 Animals

For the analysis, male Wistar rats aged 8 months were purchased from Beijing Vital River Laboratory Animal Technology Co., Ltd., Beijing, China (License No.: SCXK 2016‐00110), and housed in an SPF barrier system until 20 months of age. Male Wistar rats aged 7–8 weeks were purchased from Southern Medical University Laboratory Animal Center (License No.: SCXK 2016‐0041). All animals were handled in accordance with the Regulations on the Administration of Laboratory Animals issued by the State Science and Technology Commission of the People's Republic of China. This study was approved by the Animal Experiment Ethics Committee of Guangzhou University of Chinese Medicine.

### 2.2 Construction of CUMS models

All rats were subjected to the sucrose preference test (SPT) before the experiment. Rats with abnormal glucose consumption baseline were excluded, including those with low sucrose preference (<60%), position preference (preferring to drink liquid from a fixed position), drinking too little water (neither sucrose solution nor purified water), or consuming too much water (total water intake was more than twice the average water intake of all rats). During the SPT, all rats were housed individually.

The LOD model was established by combining natural aging with the CUMS model. After the SPT baseline, aged rats were randomly divided into the LOD group and the LUT group according to body weight and SPT results, with 12 rats in each group. Young normal rats were labeled as the CON group, with a total of 12 rats. Rats in the CON group were fed ad libitum, with a 12‐hour light/dark cycle (light time 8:00–20:00), a temperature of 23 ± 2°C, no stress was applied, and four rats were placed in each cage in Room A.

The LOD and LUT groups were subjected to chronic unpredictable mild stress, and the CUMS protocol was based on our previous study.^5^, ^18^ Rats were individually housed in Room B during the stress protocol. The stress protocol included fasting (12 h), water deprivation (12 h), continuous illumination at night (20:00–8:00 of the next day, 12 h), plantar shock (1 mA, 2 s/time, 10 times/5 min), white noise (85 dB, 5 h), night strobe illumination (300 times/min, 5 h), hot water swimming (45°C, 5 min), restraint (12 h), wet cage (10 h), and fasting and water deprivation (24 h). 1–2 stressors were applied daily in a random order, and the entire stress process lasted for 6 weeks.

### 2.3 Drug administration

**FIGURE 1:**
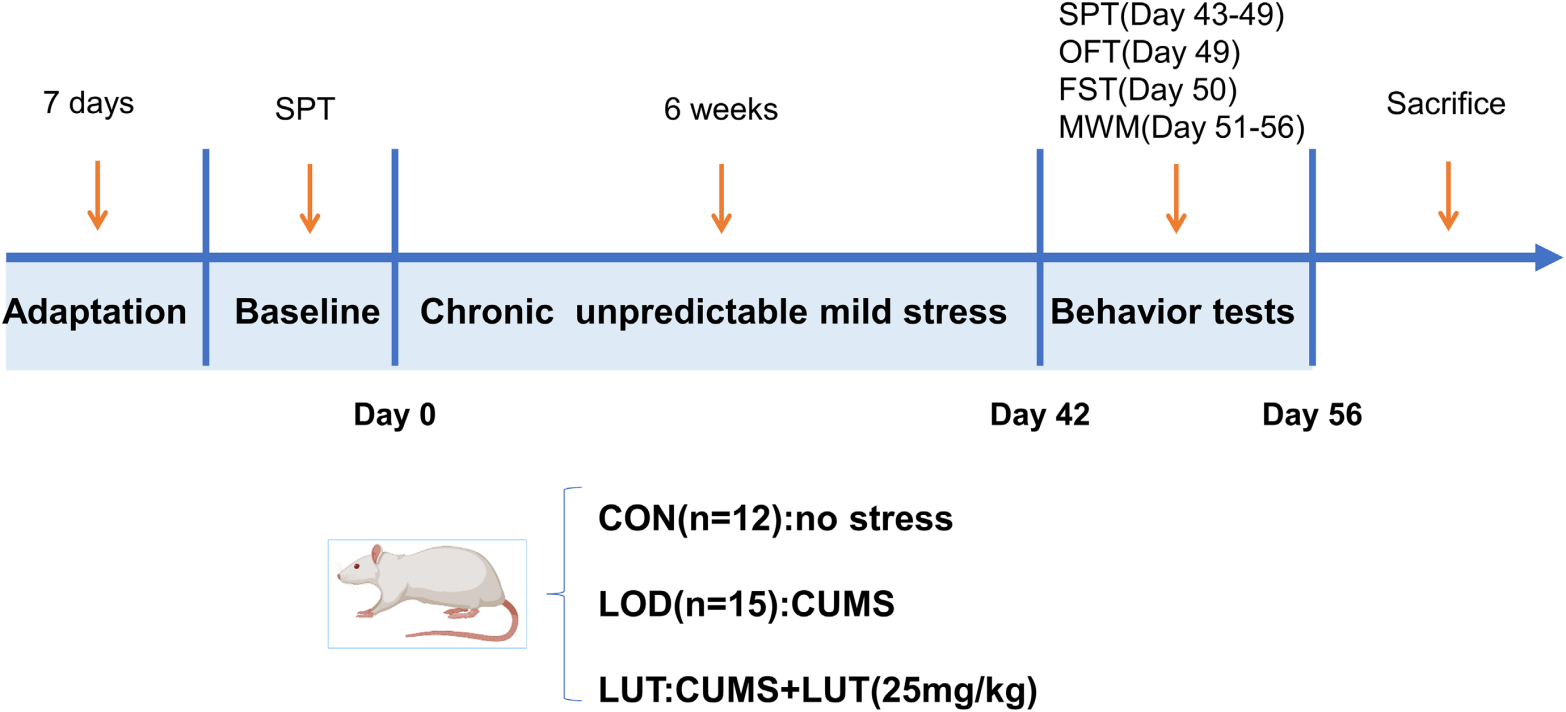
Schematic of experimental workflow. LOD, Late‐onset depression; LUT, Luteolin; CUMS, chronic unpredictable mild stress; SPT, Sucrose Preference Test; OFT, Open field test; FST, forced swim test; MWM, Morris water maze test.

Each group of rats received intragastric administration from the day of stress application for 6 weeks, and stress was discontinued at the end of 6 weeks while terminating gavage. The LUT group received intragastric administration once a day at a dose of 25 mg/kg (equivalent to twice the equivalent dose of 60 kg body weight in adults). The LUT was obtained from Nanjing Dilger Medical Technology Co., Ltd. and identified by high‐performance liquid chromatography with a purity ≥98%. The CON and LOD groups were given intragastric administration of the same amount of purified water (4 mL/kg body weight). The daily intragastric administration time points were consistent with those of the LUT group (Figure 1).

### 2.4 UPLC–MS/MS non‐targeted metabolomics analysis

Hippocampal and prefrontal cortex tissue samples (25 mg) were ground, and 100 μL of serum supernatant was extracted. Then, 400 μL of extraction solution (methanol: water = 4:1 (v:v)) was added to the hippocampus, prefrontal cortex, and serum, respectively, and vortexed and sonicated at 40 kHz for 30 min at 5°C. The samples were left at −20°C for 30 min and then centrifuged at 13,000 *g* for 15 min at 4°C. Finally, 200 μL of supernatant was pipetted for LC–MS analysis (UHPLC‐Q Exactive HF‐X system of Thermo Fisher Scientific). To assess the reproducibility of the system, QC samples were injected once every six analyzed samples throughout the analytical run.

Upon completion of loading, the LC‐MS raw data were imported into the metabolomics processing software Progenesis QI (Waters Corporation, Milford, USA) for peak identification, matching, and noise reduction via the Majorbio cloud platform (www.majorbio.com). Basic data analysis was performed using total ion chromatography, quality control analysis using principal component analysis (PCA), and partial least squares discriminant analysis (PLS‐DA) to distinguish overall differences in metabolic profiles and identify differential metabolites before and after lignocaine treatment. PLS‐DA analysis was based on metabolites of variable importance in prediction (VIP) > 1.0, and data were log10 transformed before analyzing the fold change of differential metabolites in the CON group versus the LOD group (FC). Biomarker identification was performed using the biological database HMDB (http://www.hmdb.ca/) and the Kyoto Encyclopedia of Genes and Genomes (http://www.genome.ad.jp/kegg/). The screened biomarkers were entered into the metabolic‐analyst website (https://www.metaboanalyst.ca/faces/ModuleView.xhtml) to analyze the metabolic pathways involved in the differential metabolites.

### 2.5 Enzyme‐linked immunosorbent assay

The levels of phosphatidylserine (PS, MM‐71078R1, Meimian), phosphatidylcholine (PC, MM‐71548R1, Meimian), and phosphatidylethanolamine (PE, MM‐70763R1, Meimian) were quantified using an enzyme‐linked immunosorbent assay kit (Jiangsu Meimian Industrial Co. Ltd, China). Twenty milligrams of rat hippocampus and prefrontal cortex (*n* = 5) were taken, and the tissue samples were homogenized in 1 mL of RIPA buffer (MA0151, Meilunbio) for radioimmunoprecipitation assay containing protease inhibitors. The homogenate was centrifuged at 10,000 ***g*** for 5 min at 4°C, and the supernatant was collected. The experiments were performed according to the manufacturer's instructions, and the values were assayed using an enzyme marker (3903‐2010, Bio‐rad Company, USA).

### 2.6 Molecular docking experiments

The compound names, molecular weights, and 3D structures of phosphatidylserine synthase 1 (PTDSS1), phosphatidylserine synthase 2 (PTDSS2), phosphatidylserine decarboxylase (PISD), and LUT were obtained from the PubChem database, and the corresponding 3D structures of the active ingredients were downloaded from the RCSB PDB database (http://www.rcsb.org/). The ligands and proteins required for molecular docking were prepared using AutoDock Vina software (http://vina.scripps.edu/). For the target proteins, their crystal structures underwent pretreatment, including removal of hydrogen atoms, modification of amino acids, optimization of energy, and adjustment of force field parameters. The low‐energy conformations of the ligand structures were then obtained. Finally, PTDSS2, PTDSS1, PISD, and LUT component structures were molecularly docked using Vina inside PyRx software, and their Affinity values (kcal/mol) represent the binding capacity of the ligand to the receptor. The lower the binding capacity, the more stable the binding of the ligand to the receptor. The visualization was performed using PyMOL, and the 2D plot was visualized using Discovery Studio 2020 Client.

### 2.7 Western blotting

LC3B and p62 protein expression levels were measured by western blotting. Hippocampal and prefrontal cortex tissues (*n* = 5) of rats were washed, homogenized, and lysed in RIPA buffer (MA0151, Meilunbio) containing protease inhibitors. After routine protein extraction from brain tissues, the total protein concentration was determined, and then 30 μg of total protein was mixed with 1/4 volume of 5× SDS‐PAGE sample buffer (P0015, Beyotime Biotechnology). The protein samples were boiled for 10 min, and the proteins were separated by SDS‐PAGE. After transferring the separated proteins onto PVDF membranes (IPVH 00010, Millipore), the membranes were incubated with primary antibodies, such as LC3B (1:1000, ab192890, Abcam), p62 (1:1000, 23214S, CST), and GAPDH (1:5000, 97166, CST), at 4°C overnight. Subsequently, secondary antibodies (1:5000, ab150133, Abcam) were incubated at 37°C for 2 h. The absorbance values of the protein bands were scanned using an Odyssey infrared gel imaging system (Affinity, USA). Quantitative analysis was performed using ImageJ software (version 1.45 J; National Institutes of Health, Bethesda, MD, USA).

### 2.8 Statistical analysis

Statistical analysis of the data was performed using SPSS 26.0 software (IBM, Armonk, IL, USA). The Shapiro–Wilk test was used to assess the normality of the data distribution, and the Levene test was used to assess the homogeneity of variance. For multiple group comparisons where the data met both the assumptions of normality and homogeneity of variance, one‐way ANOVA followed by Dunnett post‐hoc test was used. In cases where the assumption of homogeneity of variance was violated, the Welch test and Games‐Howell post‐hoc test were applied. For data that did not meet the normality assumption, the non‐parametric Kruskal–Wallis test for multiple independent samples was used. Two‐way repeated measures ANOVA was used to analyze the escape latency in the Morris water maze task, and if the assumption of sphericity (Mauchly's test) was violated, the Greenhouse–Geisser correction method was applied to adjust the F‐statistic and assess significance. The relationships between variables were analyzed using Spearman's rank correlation coefficient. Results are presented as mean ± SEM, with *p* < 0.05 indicating statistical significance between groups. Statistical results were plotted using GraphPad Prism 9.4.1 software (GraphPad Software, Bethesda, MD, USA).

## 3 RESULTS

### 3.1 LUT improves depression‐like behavior and enhances cognitive function of LOD rats

**FIGURE 2:**
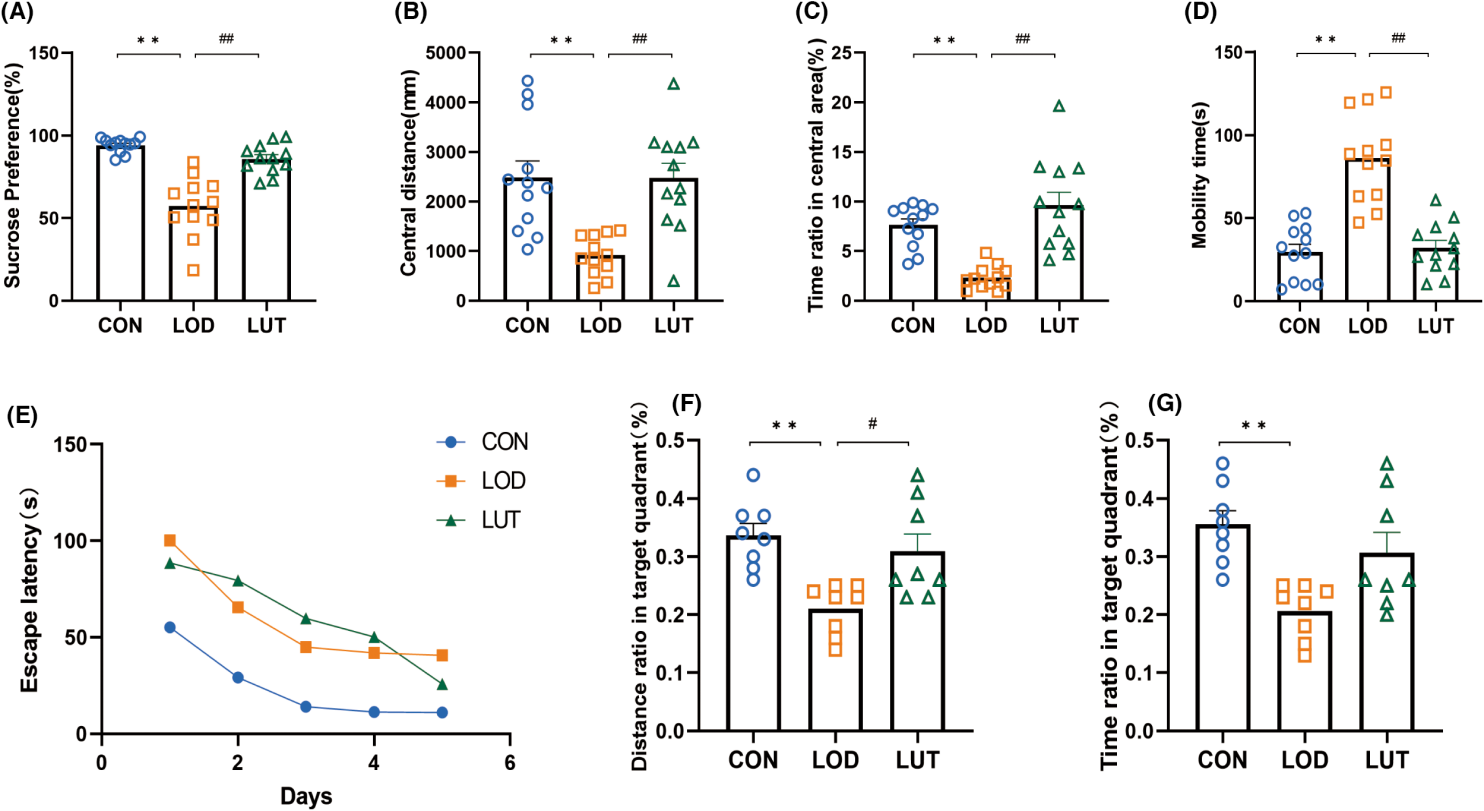
Luteolin improves depression‐like behavior in LOD rats. (A) Results of sucrose preference (%), *n* = 12; (B–C) Central region distance (mm) and central region time ratio (%) in OFT, *n* = 12; (D) Immobility time (s) in FST, *n* = 12; (E) Escape latency (s) in MWM, *n* = 8; (F–G) Target quadrant distance ratio and target quadrant time ratio (%) in MWM, *n* = 8. Data are expressed as mean ± SEM, *p < 0.05, **p < 0.01, compared with the LOD group; #*p* < 0.05, ##*p* < 0.01, compared with the LUT group.

We assessed potential anxiety and depression‐like behaviors in LOD rats using the Sucrose Preference Test (SPT), Forced Swimming Test (FST), and Open Field Test (OFT). Compared to the CON group, rats in the LOD group showed a significant decrease in sucrose preference in the SPT, as well as a significant reduction in central area distance and central area dwell time ratio in the OFT (*p* < 0.01). Additionally, they exhibited a significant increase in immobility time in the FST (*p* < 0.01). When treated with LUT, rats exhibited an increase in sucrose preference, central region distance, and time ratio (*p* < 0.01), and a decrease in immobility time (*p* < 0.01). The above results suggest that LUT reduces depression and anxiety‐like behaviors in LOD rats (Figure 2A–C).

The Morris water maze (MWM) test was used to assess the spatial cognitive abilities of LOD rats. During the place navigation experiment on days 1–5 (Figure 2E), the data conformed to Mauchly's sphericity test, revealing a decrease in platform latency between all three groups as the number of training days increased (*p* < 0.01). During the platform latency test on days 1–5, the LOD group took more time to reach the hidden platform than the CON group. In the spatial exploration test on day 6, the time and distance ratios of LOD rats in the quadrant where the target platform was located were significantly less than the number of platform traversals in CON rats (*p* < 0.01). However, the time and distance ratios of LOD rats in the quadrant where the platform was located increased after LUT treatment (Figure 2D–F). This indicates that LUT enhances learning and memory functions and improves the cognitive ability of LOD rats.

### 3.2 Metabolomic multivariate analysis of LUT treatment for LOD

#### 3.2.1 Construction of PLS‐DA model

**FIGURE 3:**
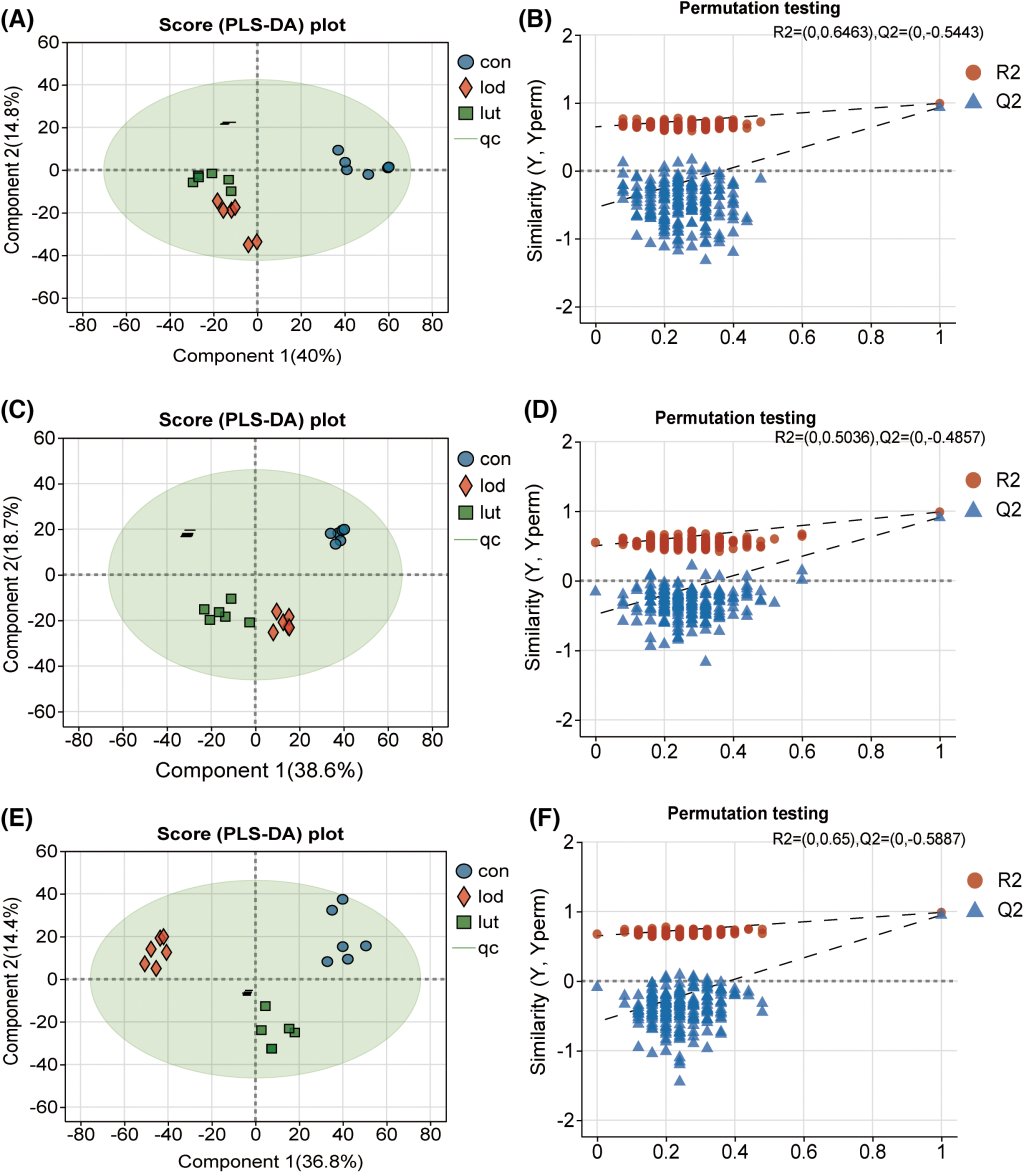
Construction of PLS‐DA models in the hippocampus, prefrontal cortex, and serum of the CON, LOD, and LUT groups. (A–B) PLS‐DA plots and model validation (R2X = 0.713, R2Y = 0.991, Q2 = 0.932) for hippocampal metabolomic features of rats in the CON, LOD, and LUT groups, *n* = 6. (C–D) PLS‐DA plots and model validation (R2X = 0.682, R2Y = 0.988, Q2 = 0.91) for prefrontal cortex metabolomic features of rats in the CON, LOD, and LUT groups, *n* = 6. (E–F) PLS‐DA plots and model validation (R2X = 0.676, R2Y = 0.986, Q2 = 0.948) for serum metabolomic features of rats in the CON, LOD, and LUT groups, *n* = 6.

Based on the partial least squares discriminant analysis (PLS‐DA), sample shifts in the hippocampus, prefrontal cortex, and serum of rats in the CON, LOD, and LUT groups showed distinct trends of cluster separation (Figure 3A–E), and the model validation was stable and non‐random (Figure 3B–F). Specifically, in the hippocampus, the overall status of differential metabolites in the LOD and LUT groups deviated from the CON group, with partial overlap of metabolites between the LUT and LOD groups. In the PLS‐DA models of the prefrontal cortex and serum, the three rat models were clearly separated from each other. These results suggest that LOD rats exhibit metabolic pattern differences between different brain regions and between central and peripheral systems, and that LUT can modulate these metabolic pattern differences in LOD rats.

#### 3.2.2 The effects of LUT on metabolic pathways and analysis of potential key metabolites in LOD rats

**FIGURE 4:**
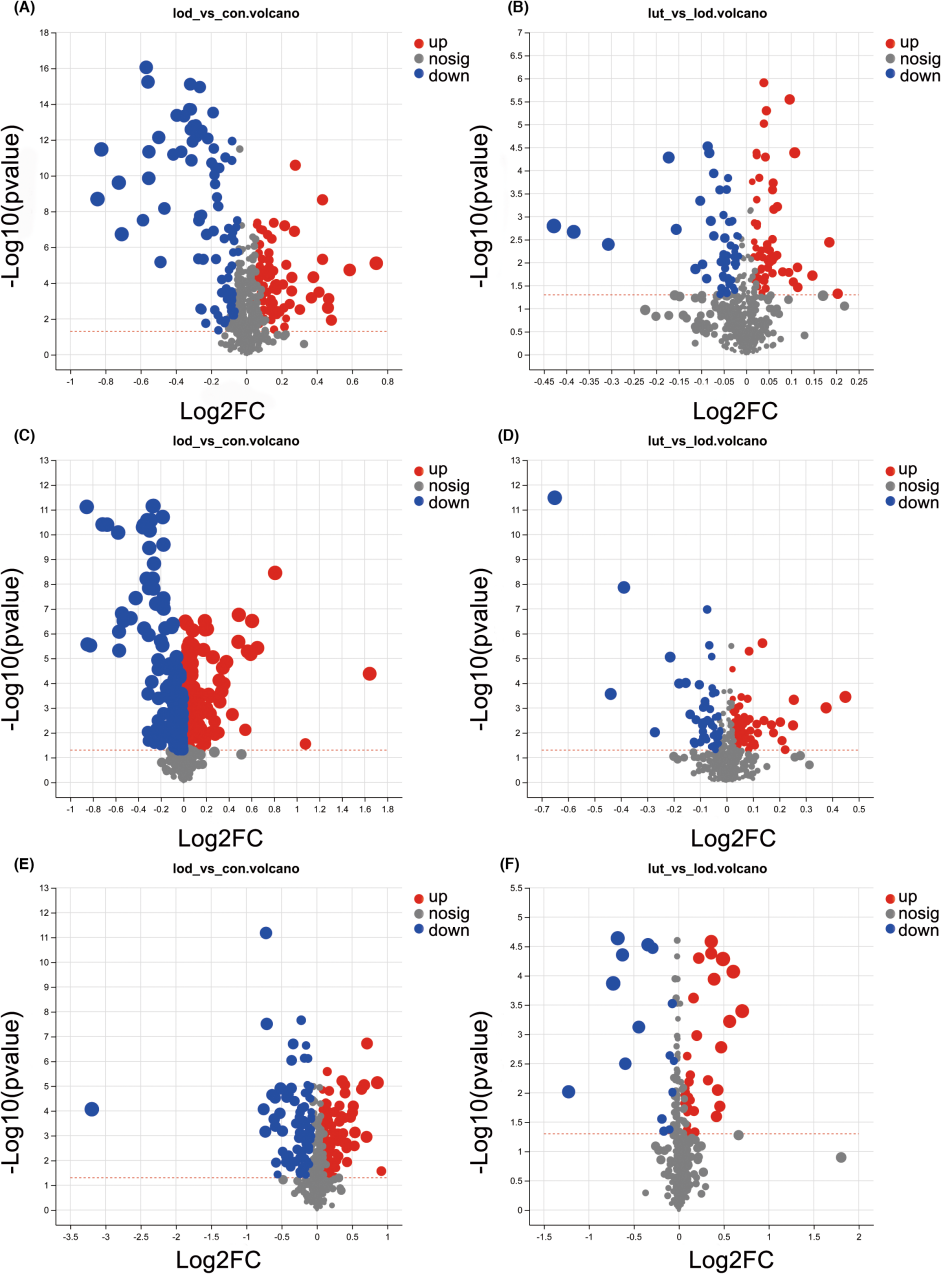
Volcano plot analysis identifies differential metabolites between groups. (A–B) Volcano plots of the hippocampal metabolites in the LOD and CON groups, and in the LOD and LUT groups, *n* = 6. (C–D) Volcano plots of the prefrontal cortex metabolites in the LOD and CON groups, and in the LOD and LUT groups, *n* = 6. (E–F) Volcano plots of the serum metabolites in the LOD and CON groups, and in the LOD and LUT groups, *n* = 6. The red dots represent upregulated endogenous metabolites, blue dots represent downregulated endogenous metabolites, and gray dots indicate no difference. The screening criteria for volcano plot analysis were *p* < 0.05 and VIP > 1.

The volcano plot (Figure 4) revealed the identification of 716, 722, and 707 metabolites in the rat hippocampus, prefrontal cortex, and serum, respectively. In comparison to the CON group, the hippocampus of LOD rats exhibited significant changes in 165 metabolites, with 76 upregulated and 89 downregulated (Figure 4A). Similarly, the prefrontal cortex showed significant changes in 189 metabolites, with 95 upregulated and 94 downregulated (Figure 4C), while the serum had significant changes in 194 metabolites, with 104 upregulated and 89 downregulated (Figure 4E). Following LUT treatment, the hippocampus of LUT rats displayed significant changes in 123 metabolites compared to the LOD group, with 68 upregulated and 55 downregulated (Figure 4B). Additionally, the prefrontal cortex exhibited significant changes in 132 metabolites, with 72 upregulated and 60 downregulated (Figure 4D), and the serum had significant changes in 42 metabolites, with 28 upregulated, and 15 downregulated (Figure 4F).

**FIGURE 5:**
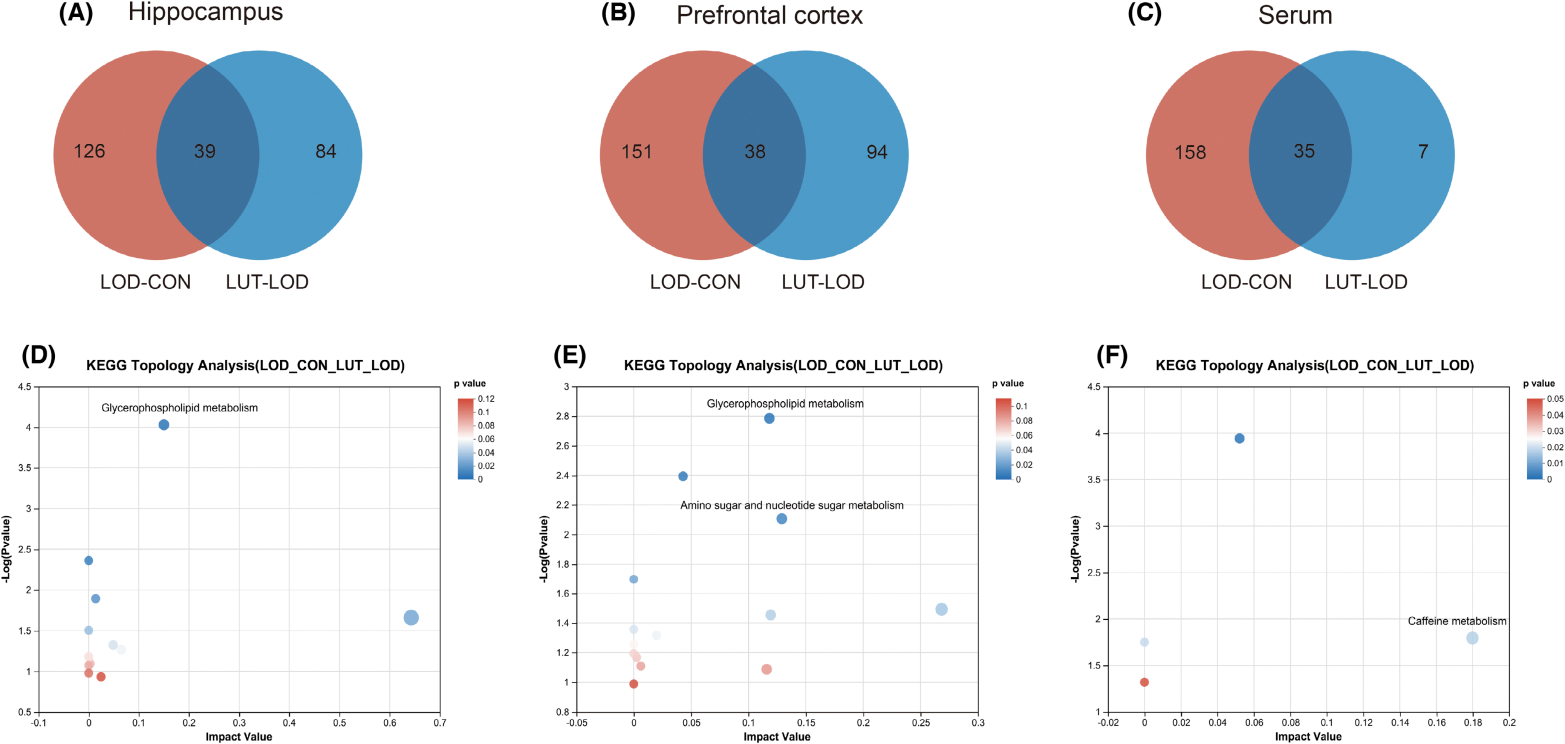
Cross‐Set and KEGG Topology Analysis of Differential Metabolites in Hippocampus, Prefrontal Cortex, and Serum to Elucidate the Effect of Luteolin on Metabolic Pathways in LOD Rats. (A–C) Venn diagrams were used to analyze the metabolic intersection of differential metabolites in hippocampus, prefrontal cortex, and serum between the LOD‐CON and LUT‐LOD groups.(D–F) Metabolic pathway analysis was performed on the common differential metabolites at the intersection of LOD‐CON and LUT‐LOD metabolism in hippocampus, prefrontal cortex, and serum. Enrichment metabolic pathway screening criteria were set at *p* < 0.05 and Impact >0.1.

We conducted a cross‐set analysis of differential metabolite sets between the LOD‐CON and LUT‐LOD groups in the rat hippocampus, prefrontal cortex, and serum. Subsequently, we performed KEGG topology analysis on the cross‐sets to investigate the metabolic pathways affected by LUT on LOD rats. Our results indicated the presence of 39 common differential metabolites between the LOD‐CON and LUT‐LOD groups in the hippocampus (Figure 5A). KEGG topology analysis of these 39 differential metabolites revealed significant and opposite alterations in the rat hippocampal glycerophospholipid metabolic pathway (Impact = 0.15, *p* = 0.000, Figure 5D) before and after LUT treatment for LOD. Similarly, there were 38 crossover metabolites between the prefrontal cortex LOD‐CON and LUT‐LOD groups (Figure 5B). KEGG topology analysis of these 38 differential metabolites revealed significant and opposite alterations in the rat prefrontal cortex glycerophospholipid metabolic pathway (Impact = 0.12, *p* = 0.012, Figure 5E) before and after LUT treatment for LOD. However, KEGG topology analysis of the 35 differential metabolites between the serum LOD‐CON and LUT‐LOD groups (Figure 5C) did not reveal any significant and opposite alterations in metabolic pathways in rat serum before and after LUT treatment for LOD.

**FIGURE 6:**
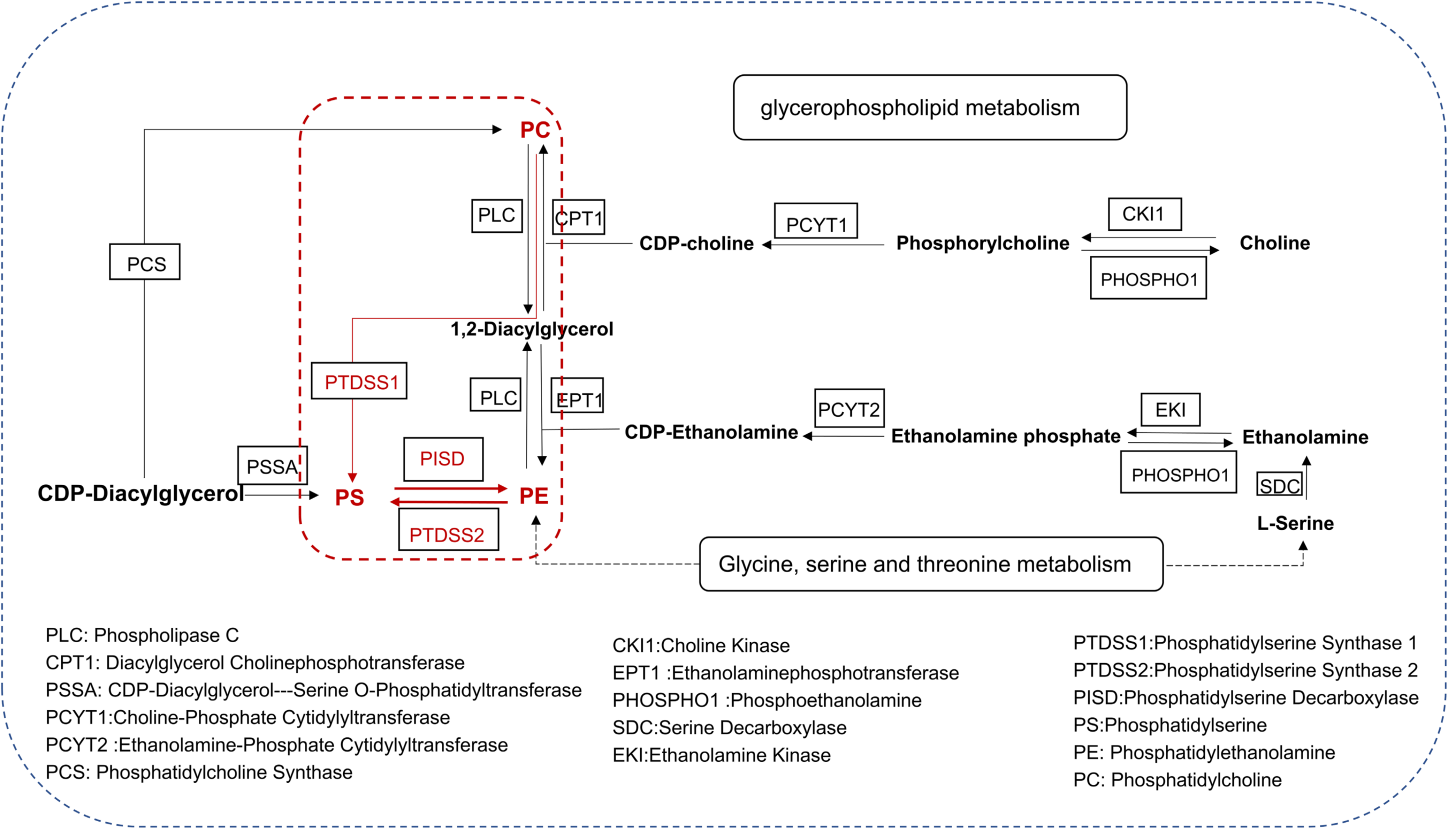
The relationship between PS, PC, and PE in the glycerophospholipid metabolic pathway.

Our findings suggest that the core drug‐acting metabolic pathway in LUT‐treated LOD rats is glycerophospholipid metabolism. We focused on metabolites associated with glycerophospholipid metabolism and combined them with metabolites significantly enriched in this pathway (Table 1). We found that PS, PC, and PE are three potential key metabolites for the effect of LUT on LOD rats. These phospholipids are associated with glycerophospholipid metabolism and can be synthesized and transformed into each other (Figure 6), as they are present in both upstream and downstream pathways of the KEGG glycerophospholipid metabolic pathway.

**TABLE 1:** Potential biological pathways of differential metabolites in the cross‐dataset of the rat hippocampus, prefrontal cortex, and serum (metabolite sets: LOD‐CON & LUT‐LOD).

Sample	Pathway Description	Pathway Impact	*p*‐Value	Metabolite
Hippocampus	Glycerophospholipid metabolism	0.150205	0.000606	PC(16:0/0:0)
				PE(15:0/22:1(13Z))
				PG(18:1(11Z)/20:4(5Z,8Z,11Z,14Z))
				PC(16:0/22:6(4Z,7Z,10Z,13Z,16Z,19Z))
				PS(18:2(9Z,12Z)/20:3(8Z,11Z,14Z))
				PE(22:4(7Z,10Z,13Z,16Z)/22:6(4Z,7Z,10Z,13Z,16Z,19Z))
Prefrontal cortex	Glycerophospholipid metabolism	0.118221	0.012303	PC(16:0/20:4(8Z,11Z,14Z,17Z))
				PE(16:0/18:1(11Z))
				PE(18:0/18:1(11Z))

#### 3.2.3 The effect of LUT differential metabolites in LOD rats

**FIGURE 7:**
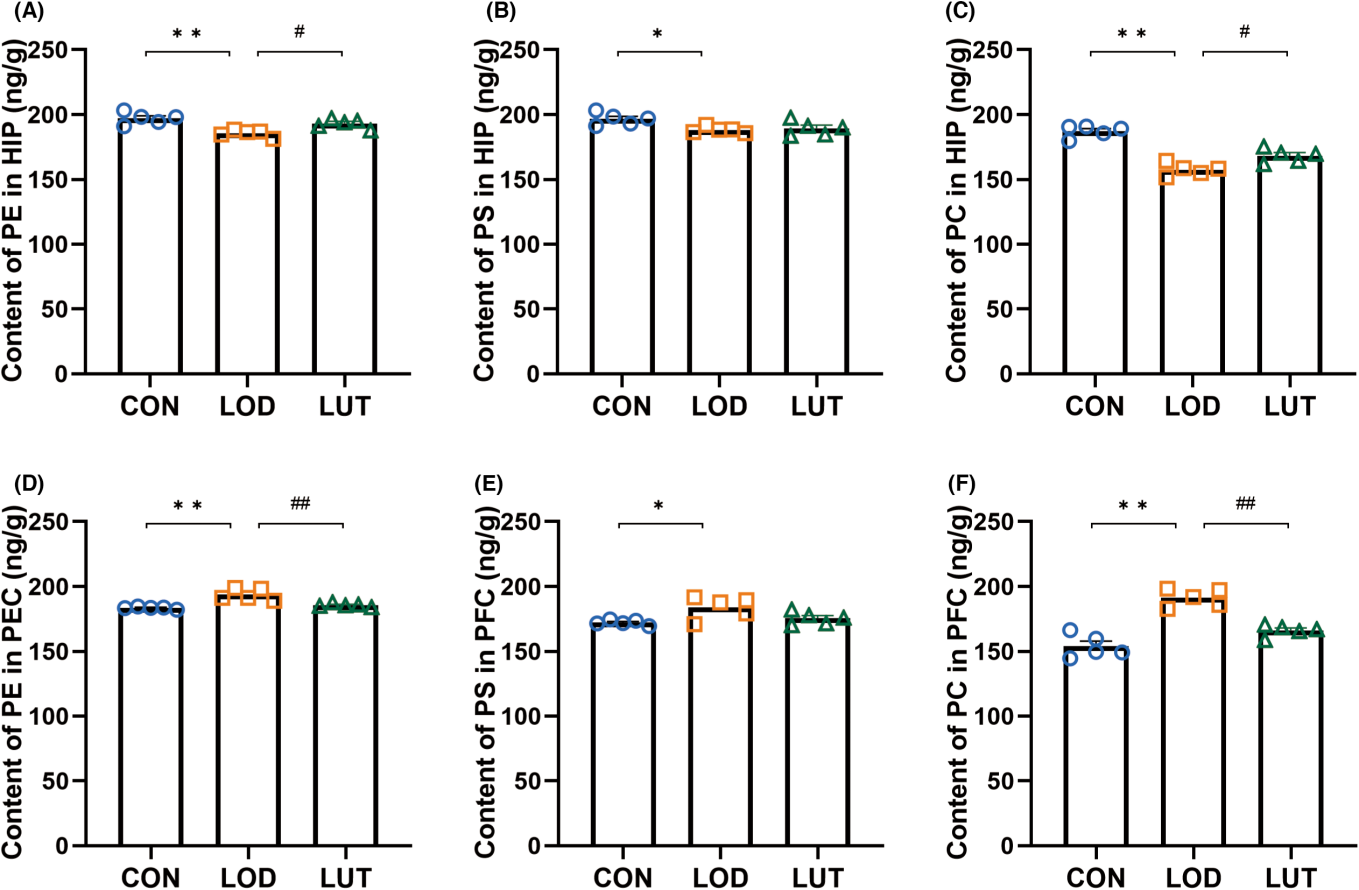
Effects of luteolin on three phospholipid metabolites (PE, PS, and PC) in the hippocampus and prefrontal cortex of LOD rats. Content expression of PE (A), PS (B), and PC (C) in the hippocampus of rats in the CON, LOD, and LUT groups (*n* = 5), and content expression of PE (D), PS (E), and PC (F) in the prefrontal cortex of rats in the CON, LOD, and LUT groups (*n* = 5). Data are presented as mean ± SEM. *p < 0.05, **p < 0.01, compared to the LOD group; #*p* < 0.05, ##*p* < 0.01, compared to the LUT group.

To validate the metabolomic results, we performed quantitative analysis of the three metabolites (PS, PC, and PE) in the rat hippocampus and prefrontal cortex using the ELISA method. Specifically, compared to the CON group, the contents of PS (*p* < 0.05), PC (*p* < 0.01), and PE (*p* < 0.01) were decreased in the hippocampus of LOD rats (Figure 7A–C), while the contents of PS (*p* < 0.05), PC (*p* < 0.01), and PE (*p* < 0.01) were significantly increased in the prefrontal cortex (Figure 7D–F). After treatment with LUT, the contents of PS, PC (*p* < 0.05), and PE (*p* < 0.05) were increased in the hippocampus of LUT rats, while the contents of PS, PC (*p* < 0.01), and PE (*p* < 0.01) were decreased in the prefrontal cortex compared to the LOD group, which was consistent with the metabolomic results. Taken together, these results fully demonstrate that glycerophospholipid metabolism disorders occur in the hippocampal and prefrontal cortex regions of LOD rats, primarily involving abnormal metabolites of PS, PC, and PE. LUT can reverse this metabolic disorder and re‐establish the dynamic balance of phospholipid metabolism in different brain regions.

#### 3.2.4 Correlation analysis between behavioral indicators and differential metabolites in metabolomics

**FIGURE 8:**
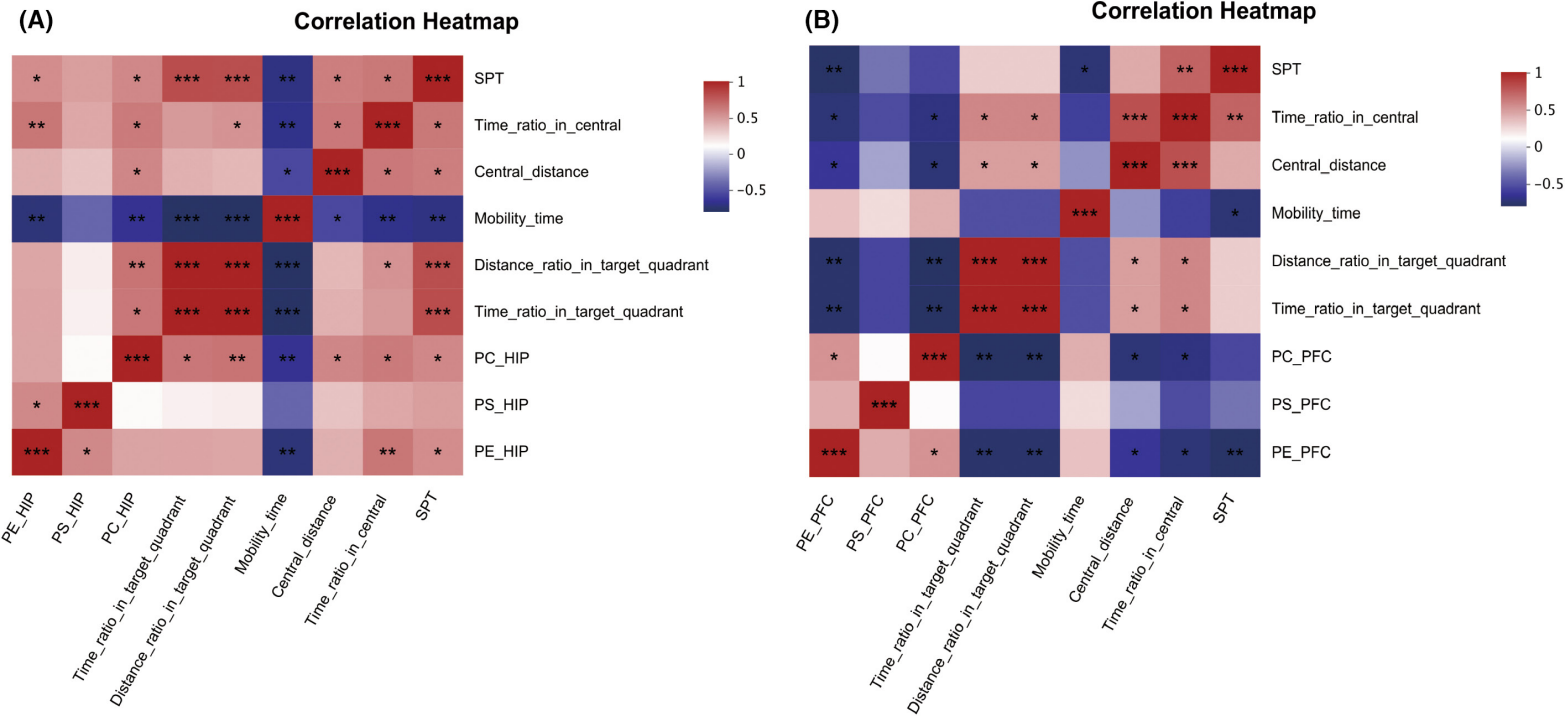
Heatmap of Spearman correlation coefficients between behavioral metrics (SPT, OFT central travel time ratio, OFT central travel time, FST immobility time, MWM station travel ratio, MWM station time ratio) and key lipid metabolites (PE, PS, and PC). (A) Correlation analysis of behavioral metrics with key lipid metabolites identified by metabolomics in the hippocampus. (B) Correlation analysis of the results of behavioral indicators in the prefrontal cortex with key lipid metabolites identified by metabolomics. The R values are displayed in different colors in the figure, with red indicating positive correlation and blue indicating negative correlation. Statistical significance is indicated by *p < 0.05 and **p < 0.01. The legend on the right shows the color intervals for different R values.

Subsequently, we performed a correlation analysis between the ELISA quantitative analysis results of PS, PC, and PE in the hippocampus and prefrontal cortex of rats and behavioral indices. The results revealed that in the hippocampus (Figure 8A), PE and PC were positively correlated with the sucrose preference in the SPT (PE: *r* = 0.546, *p* = 0.035; PC: *r* = 0.568, *p* = 0.027) and the ratio of central travel time in the OFT (PE: *r* = 0.643, *p* = 0.010; PC: *r* = 0.620, *p* = 0.014), while both PE and PC were significantly and negatively correlated with the immobility time in the FST (PE: *r* = −0.736, *p* = 0.002; PC: *r* = −0.664, *p* = 0.007). Additionally, PC was positively correlated with the central distance in the OFT (*r* = 0.571, *p* = 0.026), the ratio of platform distance in the MWM (*r* = 0.654, *p* = 0.008), and the ratio of platform time in the MWM (*r* = 0.639, *p* = 0.010). In the prefrontal cortex (Figure 8B), PE and PC were negatively correlated with the ratio of central travel time in the OFT (PE: *r* = −0.614, *p* = 0.015; PC: *r* = −0.589, *p* = 0.021), the central distance in the OFT (PE: *r* = −0.525, *p* = 0.044; PC: *r* = −0.614, *p* = 0.015), the ratio of platform distance in the MWM (PE: *r* = −0.643, *p* = 0.010; PC: *r* = −0.671, *p* = 0.006), and the ratio of platform time in the MWM (PE: *r* = −0.643, *p* = 0.010; PC: *r* = −0.671, *p* = 0.006). Additionally, PE was negatively correlated with the sucrose preference in the SPT (*r* = −0.663, *p* = 0.007). No significant correlations were observed between PS and any of the behavioral indices in either the hippocampus or prefrontal cortex.

By conducting Spearman correlation analysis, we discovered that anxiety and depression‐like behaviors in LOD rats were associated with altered glycerophospholipid metabolism. Specifically, PE and PC displayed opposite effects on anxiety, depression‐like behaviors, and cognitive abilities in the hippocampus and prefrontal cortex of LOD rats. Notably, we observed a significant positive correlation between the levels of PE and PC in the hippocampus and the despair and anxiety levels in LOD rats. Moreover, higher PC content in the hippocampus was linked to stronger spatial memory in LOD rats. In contrast, in the prefrontal cortex, both PE and PC exhibited negative correlations with anxiety and spatial memory in LOD rats, and higher PE content in the prefrontal cortex was associated with lower depression‐like behavior in LOD rats.

### 3.3 Molecular docking results of LUT and three key enzymes in the glycerophospholipid metabolic pathway

**FIGURE 9:**
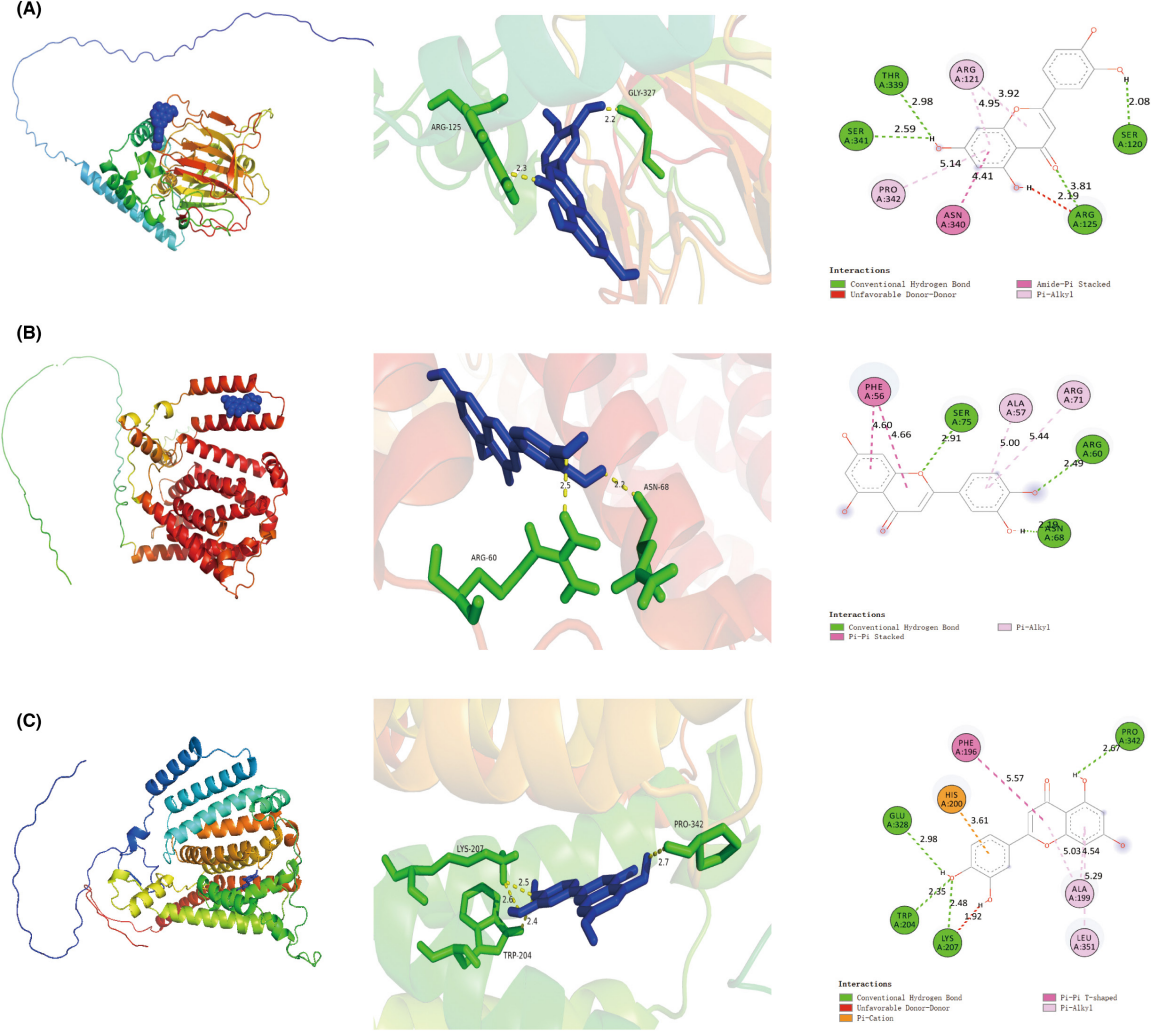
Molecular docking diagram of lignocaine with three key enzymes in the glycerophospholipid metabolic pathway. (A) PISD formed hydrogen bonding interactions with THR399, SER341, SER120, and ARG125 of lignocaine, Pi‐Alkyl interactions with ARG121 and PRO342, and Amide‐Pi stacked interactions with ASN340. (B) PTDSS1 formed a Pi–Pi stacked interaction with PHE45 of lignocaine, hydrogen bonding interactions with SER75, ARG60, and ASN68, and a Pi‐Alkyl interaction with ALA57 and ARG71. (C) PTDSS2 formed hydrogen bonding interactions with lignans GLU328, TRP204, LYS207, and PRO342, Pi‐Cation interactions with HIS200, Pi–Pi shaped interactions with PHE196, and Pi‐Alkyl interactions with ALA199 and LEU351.

PS can be generated through the base exchange of PE or PC with serine, which is catalyzed by PTDSS. PTDSS1 has a higher affinity for PC, while PTDSS2 is responsible for catalyzing PE. Additionally, PS can be converted back to PE through the action of PISD, thus forming a conversion cycle between PS, PE, and PC^29^ (see Figure 6). Building on this information, we conducted molecular docking validation of verbascoside with three key enzymes: PTDSS2, PTDSS1, and PISD (see Figure 9). The binding energies between the components and targets are presented in Table 2, which is generally considered.^30^ A docking energy value of less than −4.25 kcal/mol indicates some binding activity, less than −5.0 kcal/mol indicates good binding activity, and less than −7.0 kcal/mol indicates strong binding activity. The results revealed that verbascoside exhibited strong binding activity with PTDSS2, PTDSS1, and PISD, suggesting that these enzymes might be the targets of LUT in the treatment of LOD. However, further research and validation are necessary to confirm these findings in subsequent stages.

**TABLE 2:** Molecular docking results and binding affinity of LUT with three key enzymes in the glycerophospholipid metabolic pathway.

Compound	Target	Δ*G* (kcal/mol)
Luteolin	Phosphatidylserine synthase 1(PTDSS1)	−9.8
	Phosphatidylserine synthase 2(PTDSS2)	−7.8
	Phosphatidylserine decarboxylase (PISD)	−7.7

### 3.4 LUT bidirectionally regulates autophagy disorder in different brain regions of LOD rats

**FIGURE 10:**
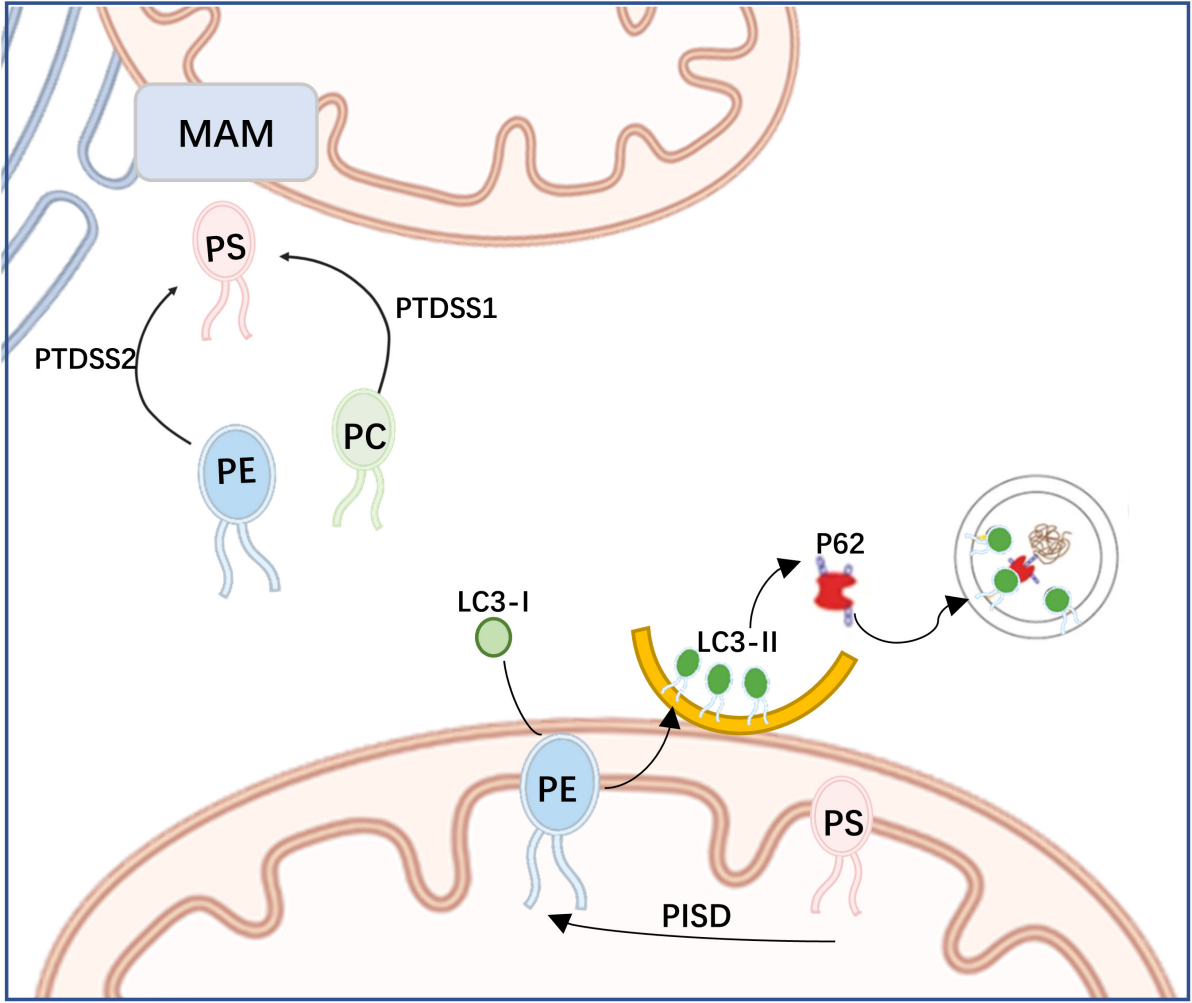
The relationship between the three phospholipid metabolites—PE, PS, and PC, and autophagy was graphed. (1) PS is produced in the ER (MAM). PSS1 catalyzes the conversion of PE to PS, while PSS2 catalyzes the conversion of PC to PS. Some newly synthesized PS is transported to the mitochondria where it is decarboxylated and converted to PE. (2) LC3‐I forms LC3‐II when bound to PE. P62 serves as a bridge between LC3B and the ubiquitinated substrate to be degraded. P62 binds to ubiquitinated proteins and delivers them to the autophagosome, which then transports them to the lysosome for degradation.

**FIGURE 11:**
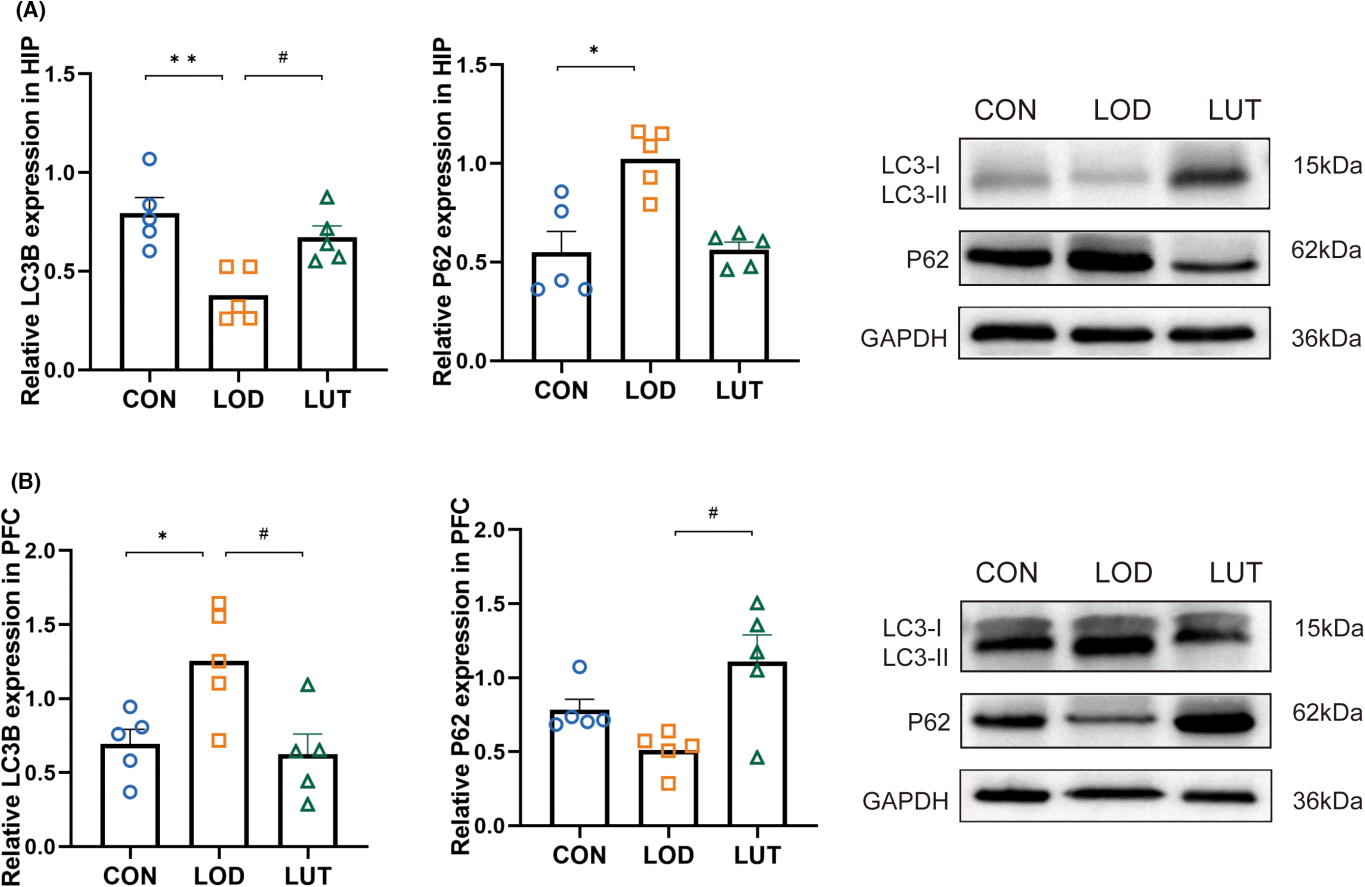
(A) Representative western blot images of LC3B and p62 protein levels in hippocampus, *n* = 5. (B) Representative western blot images of LC3B and p62 protein levels in frontal cortex, *n* = 5. Data are presented as mean ± SEM, *p < 0.05, **p < 0.01, compared with the LOD group; #*p* < 0.05, ##*p* < 0.01, compared with the LUT group.

Phospholipids are one of the main components of autophagic membrane sources, and during autophagosome formation, LC3‐I (localized in the cytosol) is covalently linked to PE and inserted into the bilayer of autophagosomes to form LC3‐II (Figure 10), so changes in phospholipids directly affect autophagosome membrane structure. We examined the protein expression levels of autophagy markers LC3B and p62 in the hippocampus and prefrontal cortex. The results showed that compared with the CON group, the hippocampal LC3II/I ratio was significantly decreased in the LOD group (*P* < 0.01), while the protein expression of p62 was increased (*P* < 0.05), suggesting that autophagic activity was decreased in the hippocampus of LOD rats (Figure 11A). Conversely, the opposite trend was observed in the prefrontal cortex, where autophagic activity was increased in LOD rats (Figure 11A). Compared with the LOD group, the LUT group had a significantly higher hippocampal LC3II/I ratio (*P* < 0.01) and correspondingly reduced protein expression of p62, while the results of autophagy indexes in the prefrontal cortex were opposite to those in the hippocampus (Figure 11B). These results indicated that the level of autophagy was disrupted in the prefrontal cortex and hippocampus of LOD rats, and LUT could exert a bidirectional regulatory effect on cellular autophagy promotion and inhibition by increasing the autophagic activity of hippocampal cells and inhibiting the autophagy of prefrontal cortex cells in LOD rats.

## 4 DISCUSSION

The complex pathogenesis of LOD renders current synthetic antidepressant treatments unsatisfactory, while LUT has shown clear therapeutic benefits in neurodegenerative diseases.^31^, ^32^ In this study, we found that LUT significantly improved depression, anxiety‐like behaviors, and cognitive deficits presented by LOD rats in behavioral experiments such as SPT, FST, OFT, and MWM. Using metabolomics techniques to evaluate the central (hippocampus, prefrontal) and peripheral (serum) metabolic profiles of LUT in LOD rats, we discovered that the antidepressant and anti‐aging effects of LUT exhibited distinct metabolic pathways in the peripheral and central systems.

Most of the key metabolites obtained from the metabolic set cross‐tabulation analysis in the hippocampus and prefrontal cortex were related to “lipid metabolism,” while no significant enrichment pathway was observed in the metabolic set cross‐tabulation analysis in peripheral serum. This indicates that LUT mainly exerts its therapeutic effect through the central nervous system of LOD rats. Studies have shown that LUT has some permeability and selectivity in the blood–brain barrier^31^ and can penetrate into the brain after oral administration. Due to the lipophilic nature of flavonoid compounds, the drug effect can accumulate in brain tissue.^33^

The brain has the highest lipid content among all body tissues, except for adipose tissue, accounting for approximately 50%–60% of the brain's dry weight.^34^ The lipid composition of the brain may influence perception and emotional behavior. The central nervous system comprises three major classes of lipids: phospholipids, sphingolipids, and cholesterol. Specifically, phospholipids and sphingolipids are closely associated with the development of neuropsychiatric disorders such as depression and anxiety disorders, and studies have demonstrated that abnormalities in lipid metabolism in the prefrontal cortex and hippocampus are linked to depression.^35^, ^36^, ^37^, ^38^, ^39^ Disturbances in lipid metabolism are also a characteristic of aging, with metabolites such as fatty acids, phospholipids, and sphingolipids affecting processes, such as inflammatory responses and oxidative stress, and being associated with aging‐related diseases.^38^, ^39^

In our study, the majority of metabolites that were significantly altered in the prefrontal cortex and hippocampal regions of LOD rats were lipid metabolites. The abnormalities in glycerophospholipid metabolism in these two brain regions showed opposite trends, which is an important aspect that has been overlooked in previous studies. Phospholipids, especially glycerophospholipids, are important components of neuronal membranes and brain phospholipids, and they also play a crucial role in regulating synaptic function.^34^, ^40^ Based on the different phospholipid glycerol backbones and polar head groups, phospholipids can be divided into different subtypes, such as PS, PE, PC, etc. PC is the most abundant glycerophospholipid in eukaryotic cell membranes, comprising 40%–50% of the cell membrane in organisms,^41^ and plays a critical role in the architecture of cell membranes.^42^ As previously mentioned, PE is a precursor of PC biosynthesis, and this type of phospholipid can affect the topology of cell membranes, thereby promoting membrane activation.^43^ PS accounts for 13%–15% of the total phospholipids in the human cerebral cortex and plays a critical role in the functioning of nerve cells.^44^

Our study found that LUT can regulate the abnormal glycerophospholipid metabolic pathway in the hippocampus and frontal lobe, mainly by affecting the levels of three phospholipid metabolites: PS, PE, and PC. Furthermore, changes in PE and PC were closely related to anxiety and depressive behaviors in LOD rats, with the accumulation of PE and PC in the hippocampus exacerbating despair and anxiety, while the accumulation of PE and PC in the frontal lobe relieved anxiety and depressive behaviors in LOD rats.

Furthermore, we found that the expression of PS, PE, and PC was significantly downregulated in the hippocampus of LOD rats. In general, a decrease in phospholipids is associated with memory loss and cognitive deficits.^45^, ^46^ However, a trend toward upregulation of the expression of these three phospholipids in the prefrontal cortex of LOD rats was observed, and LUT treatment reversed the different changes in PS, PE, and PC in the hippocampus and prefrontal cortex, respectively. Oliveira^47^ found that chronic stress led to region‐specific changes in the lipidome of depressed rats, with the highest degree of changes in lipid metabolism in the prefrontal cortex compared to the hippocampus. Our study found that phospholipid content does not always show the same changes in different regions of the brain, and that many age‐related neurochemical changes can be traced to structural and functional alterations in neuronal membranes.^48^ As mentioned previously, phospholipids are one of the main components of autophagic membrane sources, and cell culture experiments have shown that exposure to PE results in the loss of asymmetric amino phospholipids in cell membranes, which can promote cell apoptosis and autophagy.^49^ We speculate that the opposite abnormal metabolic changes of phospholipids in different brain regions may be associated with abnormal alterations in autophagy.

Autophagy is an evolutionarily conserved lysosomal‐mediated biodegradation process with an important role in regulating intracellular homeostasis. LC3B is a common marker of autophagic membranes, which can be converted to LC3‐I through a series of ubiquitin‐like reactions and hydrolyzed to form LC3‐II, which is then stabilized in the autophagic inner and outer membranes. LC3‐II/I is a marker for detecting autophagic activity.^50^ P62 is the most important cargo protein for selective autophagy. LC3‐II can further form a complex with p62 protein and participate in lysosomal degradation.^51^

In our study, compared with the CON group, the LC3‐II/I ratio was significantly reduced in the hippocampus of LOD rats, and the expression of p62 protein was significantly increased, indicating that the autophagic activity of the hippocampus of LOD rats was reduced. In contrast, in the prefrontal cortex, cellular autophagy was activated in LOD rats, and LUT treatment inhibited cellular autophagy in the prefrontal cortex of LOD rats. This result is consistent with our metabolomic experimental study, in which glycerophospholipid metabolism was disturbed in the hippocampus and prefrontal cortex of LOD rats, and accordingly, autophagy levels were disturbed in these brain regions. Previous studies^52^ have also found that lipopolysaccharides can lead to disturbed autophagy levels in the cortical and hippocampal regions of the rat brain, and the mechanism by which antidepressant drugs act may involve bidirectional regulation of autophagy levels in different regions of the brain and body.^53^ The bidirectional regulation of autophagy in different brain regions may play a crucial role in the therapeutic effects of LUT on LOD rats, as autophagy is intimately linked to various cellular processes, including lipid metabolism and synaptic function.^34^, ^40^ However, further research is needed to fully elucidate the precise mechanisms underlying the effects of LUT on autophagy in LOD rats.

Autophagy is a crucial aspect of the stress response and is generally considered a beneficial process; thus, age‐related neurodegenerative diseases may delay or ameliorate disease onset through chronic induction of autophagy.^54^, ^55^ However, excessive activation of autophagy may be detrimental under certain conditions, and inhibition of autophagy has been shown to attenuate the induction of depression‐like behavior in rats by lipopolysaccharides, while also reducing levels of neuroinflammation.^52^ Therefore, bidirectional regulation of depression by autophagy may exist, and trends in autophagy levels may not always be consistent across different brain regions. In our study, autophagy in the hippocampus of LOD rats was suppressed, while activation of autophagy levels was observed in the prefrontal cortex. One study found that the maternal separation stress model also induced different autophagic responses in the hippocampus and prefrontal cortex,^56^ which is consistent with our results.

Additionally, LUT can regulate autophagy disorders by modulating phospholipid metabolism between different brain regions. The role of autophagy in antidepressant action can usually be explained by maintaining protein homeostasis, particularly the functional integrity of membrane proteins involved in synaptic neurotransmission.^57^ It has been shown that PS,^29^ PE,^58^ and PC^59^ play a role in the regulation of synapse formation, synaptic transmission, and synaptic plasticity. Due to changes in the aforementioned glycerophospholipid metabolism, metabolic dynamics may affect mitochondrial autophagy programs. Therefore, we hypothesize that LUT may alter synaptic neurotransmission by changing the integrity of cell membrane organization through autophagy, and further research is required to elucidate the exact physiological role of metabolites in the regulation of mitochondrial autophagy. Understanding these complex interactions between autophagy, phospholipid metabolism, and synaptic function could provide valuable insights into the therapeutic potential of LUT in treating depression and age‐related neurodegenerative diseases.

At the same time, our study has some limitations: (1) We described differences in the metabolic pathways between the central and peripheral systems of LUT action in LOD rats, and further investigation of the causes of such metabolic differences is needed to clarify the pharmacokinetics of LUT in the peripheral blood system and the central nervous system. (2) We found abnormal changes in glycerophospholipid metabolism in the hippocampus and prefrontal cortex of LOD rats, and molecularly docked three key enzymes in the glycerophospholipid metabolic pathway, which may be the drug targets of LUT for LOD treatment. It is necessary to further validate these targets at the molecular level and to use lipid‐targeted metabolomics techniques for more precise qualitative and quantitative analysis of lipid metabolites.

## 5 CONCLUSION

Our study demonstrates that LUT can ameliorate depression‐like behavior and cognitive deficits in LOD rats. We also observed abnormalities in glycerophospholipid metabolism in the hippocampal and prefrontal cortex brain regions of LOD rats, especially in PS, PE, and PC, which show metabolic alterations in different brain regions. LUT can bidirectionally regulate disorders of glycerophospholipid metabolism in the hippocampus and prefrontal cortex of LOD rats, thus improving autophagy disorders. These findings provide new perspectives for further investigating the pathophysiology of LOD and may help identify potential targets for the anti‐LOD effects of LUT.

## AUTHOR CONTRIBUTIONS

L‐LW and CY designed and supervised the study and revised the manuscript. X‐FW and H‐FX analyzed the data and wrote the manuscript. N‐XZ, H‐ZL, G‐LY, and K‐GL performed experiments. All authors gave final approval of the manuscript to be published.

## FUNDING INFORMATION

This work was supported by the National Natural Science Foundation of China (grant number: 82174255, to Lili Wu).

## CONFLICT OF INTEREST STATEMENT

The authors declare that they have no known competing financial interests or personal relationships that could have appeared to influence the work reported in this paper.

## Supporting information


Data S1

## Data Availability

The data that support the findings of this study are available from the corresponding author upon reasonable request.
