# Supplementary material for: Luteolin alleviates depression‐like behavior by modulating glycerophospholipid metabolism in the hippocampus and prefrontal cortex of LOD rats
Source: CNS Neurosci Ther. 2023 Sep 16;30(3):e14455. doi: 10.1111/cns.14455 (PMC10916417; doi:10.1111/cns.14455)
Supplement: Supplementary file 1 — Data S1 [file CNS-30-e14455-s001.pdf]

Supplementary Information:
Includes Supplementary Methods and Supplementary Tables

Supplementary Materials and Methods:

Behavioral Tests

Sucrose Preference Test(SPT)

The SPT consisted of four phases: 1. Two-bottle training lasting 48 hours, during which rats were fed freely. 2. Baseline test conducted three times, with each test lasting 12 hours and an interval of 12 hours between tests. The baseline sugar water preference degree was recorded after each test. Food deprivation occurred during baseline testing, and rats were allowed to eat freely during non-testing periods. 3. Fasting and water deprivation lasting 24 hours. 4. Sucrose preference test lasting 12 hours, during which rats were deprived of food. During the two-bottle training, baseline test, and sucrose preference test, rats were given equal amounts of pure water and 1% sucrose solution, and sucrose preference was calculated after 12 hours of free drinking. Sucrose preference was calculated as follows: Sucrose preference (%) = Sucrose solution consumption/Total liquid intake × 100%.

Open Field Test(OFT)

The OFT is mainly used to evaluate anxiety by studying spontaneous activity, exploratory learning, and other behaviors of rats. Rats were placed in an open, quiet, and dark behavioral room 1 hour before the start of the experiment to familiarize themselves with the room environment. At the beginning of the experiment, the operator gently placed the rat in the center of the box (100 cm × 100 cm × 48 cm) by pinching the tail 2/3 from the root and then quickly left the position of the open field box. After the rats were accustomed to the box for about 10 seconds, the analysis system recorded the total distance and the number of times the rats moved within 5 minutes to evaluate the spontaneous activity of the rats. It is important to note that the excreta of the rats need to be cleaned at the end of each test. Then, the odor left by the previous rat is removed with bromogeramine and 75% alcohol, and the enclosure is allowed to dry. During the test, it is necessary to maintain a quiet environment, avoid light and noise, and prevent personnel interference.

Forced Swimming Test(FST)

The FST is primarily used to assess despair behavior in rats. One hour prior to the experiment, the rats were moved into the room where the apparatus is located to acclimate to the experimental environment, and the order of the rats in each group was randomized. Each rat was placed in a cylindrical container

with a diameter of 30 cm, a height of 100 cm, and a water capacity of 35 cm (at 25±1°C), and was forced to swim. The behavior of the rat during a 6-minute period was recorded using a camera, while the operators, who were blinded to the treatment groups, recorded the duration of immobility during the final 4 minutes. The duration of immobility in rats was defined as the length of time during which the rat remained motionless with its limbs still or with only slight movements of the forepaws and tail, leading to the head protruding above the water surface.

Morris Water Maze Test(MWM)

The MWM was used to assess learning and spatial memory functions in rats. The MWM consisted of two main phases: the localization navigation test and the spatial search experiment, which lasted for a total of 6 days, during which the water temperature was maintained at 25°C. The water pool was divided equally into four quadrants, with the platform located in the center of any quadrant. A camera with a display system was placed above the maze to record the rat's movements simultaneously.

The localization navigation test was used to test the rats' ability to acquire learning and memory for the water maze and lasted for 5 days, with four training sessions per day, each 30 minutes apart. The training was conducted by randomly selecting one quadrant as the water entry point and placing the rat into the water. The rat was allowed to stay for 10 seconds when it climbed to the hidden station, and if it did not find the station within 120 seconds, the rat was guided to the platform for 10 seconds. The latency of the rat to find the station was recorded by the image tracking system during the experiment. The spatial search experiment was used to measure the ability of the rats to retain the memory of the spatial location of the platform. On day 6, the concealed platform was removed, and the rats were placed facing the wall of the pool at any one entry point. The time spent in the platform quadrant, the number of crossings over the previous platform location, and the percentage of time spent in the target quadrant were recorded.

Supplementary tables:

Supplementary Table 1A: Differential Metabolites of LUT-LOD in the Rat Hippocampus

Metabolite	VIP_pred_OPLS-DA	FC	P_value	FDR	Retention time	M/Z
PC(16:0/0:0)	1.554143897	1.016279	0.0000464	0.008472	6.981233333	518.3222435

PE(15:0/22:1(13Z))	1.885528603	1.029909	0.0000509	0.008472	7.364633333	804.5759
PG(18:1(11Z)/20:4(5Z,8Z,11Z,14Z))	1.015197332	1.012303	0.000622	0.01411	7.57215	831.4915
PC(16:0/22:6(4Z,7Z,10Z,13Z,16Z,19Z))	1.130942709	1.013626	0.005203	0.04859	8.532666667	850.5598
PS(18:2(9Z,12Z)/20:3(8Z,11Z,14Z))	1.180356705	1.013141	0.00007716	0.004564	7.486766667	830.4953
PE(22:4(7Z,10Z,13Z,16Z)/22:6(4Z,7Z,10Z,13Z,16Z,19Z))	1.000026569	1.010334	0.001961	0.02706	7.391616667	838.539

Supplementary Table 1B: Differential Metabolites of LOD-CON in the Rat Hippocampus

Metabolite	VIP_pred_OPLS-DA	FC	P_value	FDR	Retention time	M/Z
PC(16:0/0:0)	1.404916	0.866654	0.01848	0.05021	6.981233333	518.3222435
PE(15:0/22:1(13Z))	1.15589	0.964499	0.00000003136	0.00000003136	7.364633333	804.5759
PG(18:1(11Z)/20:4(5Z,8Z,11Z,14Z))	1.083545	0.947271	0.000411	0.001745	7.57215	831.4915
PC(16:0/22:6(4Z,7Z,10Z,13Z,16Z,19Z))	1.348769	0.943732	0.0000002556	0.000004551	8.532666667	850.5598
PS(18:2(9Z,12Z)/20:3(8Z,11Z,14Z))	1.130753	0.949484	0.000001936	0.00002478	7.486766667	830.4953
PE(22:4(7Z,10Z,1	1.452405	0.907773	0.00006323	0.000392	7.391616667	838.539

3Z,16Z)/22:6(4Z,7Z,10Z,13Z,16Z,19Z))

Supplementary Table 2A: Differential Metabolites of LUT-LOD in the Rat Prefrontal Cortex

Metabolite	VIP_pred_OPLS-DA	FC	P_value	FDR	Retention time	M/Z
PC(16:0/20:4(8Z,11Z,14Z,17Z))	1.490666	0.971055	0.01067	0.08031	8.722283333	782.5699
PE(16:0/18:1(11Z))	3.246634	0.862312	0.000008812	0.001934	7.89175	716.5232
PE(18:0/18:1(11Z))	2.816822	0.898355	0.00009741	0.007971	8.035133333	726.5443

Supplementary Table 2B: Differential Metabolites of LOD-CON in the Rat Prefrontal Cortex

Metabolite	VIP_pred_OPLS-DA	FC	P_value	FDR	Retention time	M/Z
PC(16:0/20:4(8Z,11Z,14Z,17Z))	1.704955	1.06147	0.000007465	0.00012	8.722283333	782.5699
PE(16:0/18:1(11Z))	1.191599	1.04888	0.000908	0.004922	7.89175	716.5232
PE(18:0/18:1(11Z))	2.820706	1.27309	0.00004833	0.000516	8.035133333	726.5443
